# Simultaneous Presence of Antibodies against Five Respiratory Pathogens in Unvaccinated Dairy Calves from South-Western Poland

**DOI:** 10.3390/ani14172520

**Published:** 2024-08-30

**Authors:** Agnieszka Lachowicz-Wolak, Małgorzata D. Klimowicz-Bodys, Katarzyna Płoneczka-Janeczko, Michał Bednarski, Kamil Dyba, Piotr Knap, Krzysztof Rypuła

**Affiliations:** 1 Division of Infectious Diseases of Animals and Veterinary Administration, Department of Epizootiology and Clinic of Birds and Exotic Animals, Faculty of Veterinary Medicine, Wroclaw University of Environmental and Life Sciences, pl. Grunwaldzki 45, 50-366 Wroclaw, Poland; malgorzata.klimowicz-bodys@upwr.edu.pl (M.D.K.-B.); katarzyna.ploneczka-janeczko@upwr.edu.pl (K.P.-J.);; 2 Department of Applied Mathematics, Wroclaw University of Environmental and Life Sciences, pl. Grunwaldzki 45, 50-366 Wroclaw, Poland; kamil.dyba@upwr.edu.pl; 3 “Epi-Vet” Veterinary Diagnostic Laboratory, Faculty of Veterinary Medicine, Wroclaw University of Environmental and Life Sciences, pl. Grunwaldzki 45, 50-366 Wroclaw, Poland; piotr.knap@upwr.edu.pl

**Keywords:** BRD, BoAHV1, BPIV3, BVDV, BRSV, *M. bovis*, dairy cattle seroprevalence

## Abstract

**Simple Summary:**

Bovine Respiratory Disease (BRD) is a major concern for cattle welfare globally, causing respiratory issues and significant economic losses. This study investigated the presence of antibodies against five BRD pathogens in calves from south-western Poland. Researchers tested serum samples from 4095 dairy calves, aged 7–12 months, that recently showed BRD symptoms and had never been vaccinated. They found that 47.08% of the herds had antibodies against at least one of the tested pathogens. The overall prevalence rates were as follows: BoAHV1 (21.54%), BVDV (32.0%), BRSV (34.15%), BPIV3 (34.31%), and *M. bovis* (31.38%). Notably, BRSV and BPIV3 antibodies often appeared together in serum samples. This study underscores the importance of preventive measures against these pathogens to protect cattle health and reduce economic losses in the region.

**Abstract:**

Bovine Respiratory Disease (BRD) poses a significant threat to cattle welfare worldwide, affecting their respiratory system and causing substantial economic losses. BRD is multifactorial in nature. This research aimed to investigate the serological profile of calves for the five main bovine respiratory pathogens. Serum samples were collected from dairy calves aged 7–12 months that had never been vaccinated against tested pathogens and had recently shown signs of BRD. A total of 4095 calves from 650 dairy herds located in south-western Poland were examined. The Commercial Indirect Respiratory ELISA Kit Multiplexed—Double well—BIO K 284/5 (Bio-X Diagnostics, Rochefort, Belgium) was used to determine the presence of antibodies against BVDV, BoAHV1, BRSV, BPIV3, and *M. bovis*. The presence of antibodies against at least one of the tested pathogens was found in 306 (47.08%) herds. The overall prevalence of antibodies was as follows: BoAHV1 21.54%, BVDV 32.0%, BRSV 34.15%, BPIV3 34.31%, and *M. bovis* 31.38%. The strongest correlation was between BRSV antibodies positive sera and BPIV3 antibodies positive sera. Among the five pathogens tested, antibodies to BVDV, BRSV, BPIV3, and *M. bovis* were most commonly detected simultaneously. The results of this study indicate the need for preventive measures against these pathogens in the studied region.

## 1. Introduction

Bovine Respiratory Disease (BRD) is spread globally and causes high mortality—especially among calves under 12 months of age—as well as economic losses due to the lower milk yield of cows with a history of respiratory disease [1,2,3,4]. BRD is multifactorial in nature; it is a disease with multiple causes, including viral and bacterial infections, and in some cases can be exacerbated by environmental factors and stress [5,6,7]. Pathogens considered to contribute to BRD include the following: *Pasteurella multocida* (*P. multocida*), *Mannheimia haemolytica* (*M. haemolytica*), *Mycoplasma bovis* (*M. bovis*), *Histophilus somni* (*H. somni*), bovine parainfluenza virus type 3 (BPIV3), bovine respiratory syncytial virus (BRSV), bovine coronavirus (BCoV), bovine viral diarrhea virus (BVDV), bovine alphaherpesvirus type 1 (BoAHV1), bovine adenovirus (BAdV), and influenza D virus (IDV) [8,9,10,11,12,13,14,15,16,17]. However, the number of pathogens potentially involved in the pathogenesis of BRD is significantly larger, as demonstrated by metagenomic analysis [18]. The multiplicity of pathogens contributing to BRD complicates infection monitoring and the provision of anti-infection prophylaxis. Performing tests on a herd-by-herd basis entails an additional diagnostic cost for herd owners. Therefore, the periodic performance of epidemiological studies can contribute to the local conduct of better-tailored prophylaxis to reduce mortality and morbidity among calves and, consequently, to increase the productivity of dairy cows in the future [19]. Studies on the cooccurrence or correlation of pathogens in BRD contribute to developing customized prophylaxis plans for dairy cattle farms in the region. They may also potentially influence the creation of better-tailored multivalent vaccines [8,18,19,20,21].

In south-western Poland, the last comprehensive study on BRD epidemiology was conducted in the mid-1990s; therefore, it was decided to update the knowledge on the subject [22]. Pathogens recognized as primary causes of BRD (BRSV, BPIV3, BoAHV1, and BVDV) have been included in this study [16,20]. Commercial laboratory tests detecting the presence of antibodies against these agents are available, and vaccines for all studied viruses can be found in the European Union. Additionally, considering that mycoplasmosis is a frequent and serious bacterial complication of BRD, a test detecting antibodies against the previously mentioned viral pathogens and *M. bovis* has been selected [8,20,23]. It was decided to examine a group of calves due to their greater susceptibility to BRD compared to older cattle. The chosen group of calves, which had previously exhibited symptoms of BRD and were aged 7 to 12 months, had not received any vaccinations against the studied pathogens. At this age, maternal antibodies should no longer be present in their systems [24,25]. This study aimed to determine the seroprevalence and simultaneous presence of antibodies to five known BRD-causing pathogens—BRSV, BPIV3, BoAHV1, BVDV, and *M. bovis*—in unvaccinated calves from herds located in south-western Poland.

## 2. Material and Methods

### 2.1. Sample Collection

#### 2.1.1. Samples

This study included samples taken between September 2018 and September 2022. Blood samples were collected aseptically from the jugular vein of each calf using 5 mL anticoagulant-free vacuum blood collection tubes and transported under refrigerated conditions, i.e., 4 °C to the “Epi-Vet” veterinary diagnostic laboratory of Wroclaw University of Life Sciences within 24 h. Serum was separated by centrifugation at 3000× *g* for 10 min at room temperature. Haemolysed samples or those containing coagulum were not tested. The resulting sera from individual calves, with a minimum of six calves, were pooled to obtain a single result from the herd. Pooling of samples was performed by combining, in one sterile tube, 100 µL of each serum taken from calves from a given herd. The samples thus pooled were mixed and constituted one total serum sample from the herd for further testing. The sera were tested on the same day, or if it was necessary to store them and perform the test on another day, they were frozen and preserved at −20 °C.

#### 2.1.2. Calves

Material was collected in cooperation with practitioners providing veterinary care for tested herds. Samples were collected by these veterinarians from calves aged 7–12 months that had never been vaccinated against the tested pathogens. The study group consisted of calves that had recently shown signs of BRD such as nasal and ocular discharge, fever, cough, increased respiratory rate, increased lung field murmur, respiratory wheezing, and rales. The calves were post-BRD or in the midst of chronic disease, but the first symptoms occurred no less than 3 weeks before the material was collected for testing.

#### 2.1.3. Herds and Region

**Figure 1:**
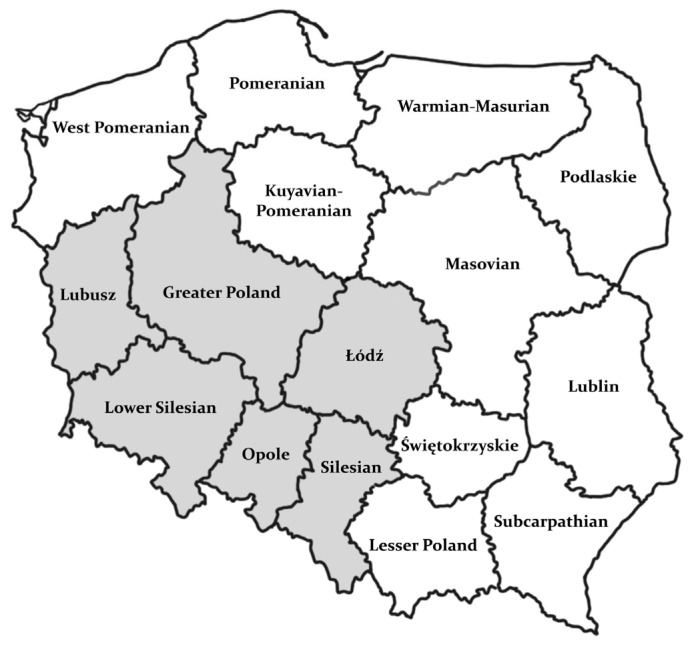
Map of Poland with regions marked in gray where tested dairy cattle herds were located.

The material came from dairy cattle herds located in south-western Poland (Figure 1). In the surveyed herds, which consisted of at least 50 animals, BRD was noted, and no vaccination against the examined pathogens was carried out.

### 2.2. Tests

The Commercial Indirect Respiratory ELISA Kit Multiplexed—Double well—BIO K 284/5 (Bio-X Diagnostics, Rochefort, Belgium) was used to determine the presence of antibodies against BVDV, BoAHV1, BRSV, PI-3V, and *M. bovis*. The ELISA procedure was performed according to the manufacturer’s instructions. Optical density was measured at 450 nm using an ELISA plate reader (BioTek Instruments, Winooski, VT, USA). According to the manufacturer’s instructions and the table provided, the determination of serum positivity (SP) as a plus sign (+) was performed (Table 1). A sample was considered positive if it yielded a result greater than or equal to one plus sign (+).

### 2.3. Statistical Analysis

#### 2.3.1. Sample Size Calculations

Based on the literature data on the frequency of appearance of antibodies to the tested pathogens among cattle in Poland, it was determined that in order to obtain reliable results, the minimum number of examined farms should be 369 [22,26,27,28,29]. To perform the calculations the following formula was used (at a confidence level of 0.95) [30]:

n = [1.96^2^ × P_exp_ × (1 − P_exp_)]/d^2^

- n—required sample size;
- P_exp_—expected prevalence;
- d—desired absolute precision.

The number of samples per herd was determined based on the formula (at a confidence level of 0.95) [30]:

n = [1 − (1 − p_1_)^1/d^] × [N − (d/2)] + 1

- n—number of samples from the herd;
- p_1_—probability of detecting at least one infected calf;
- N—number of animals in the herd;
- d—minimum number of affected animals expected in the herd (N × expected prevalence).

It was calculated that an average of 5.03–5.2 calves should be tested from one herd, thus, the results when material from one herd came from a minimum of six calves were eligible for testing.

#### 2.3.2. Results Analysis

The unit analyzed in this study was the herd. The seroprevalence means the percentage of herds with the presence of antibodies against the tested pathogen. Ninety-five percent confidence intervals (CI 95%) were calculated using the Wilson score method.

In this article, combinations without repetitions of occurrences of antibodies to individual pathogens are called configurations, and the number of possible combinations was calculated by the binomial coefficient.

Correlations of the presence of antibodies against tested pathogens were shown using Spearman’s rank order coefficient, for which α = 0.05 and a confidence level of *p* = 0.95 were adopted, and a *p*-value of <0.001 was adopted as the significance level. The serum positivity rate (SP) constituted ranks it was assumed “+” as weakly positive, “++” and “+++” as moderately positive, and “++++” and “+++++” as strongly positive. According to the basic principles of statistics, in the analysis of Spearman’s rank correlation coefficient, the sign of the coefficients was evaluated, interpreting negative as negative correlation and positive as positive correlation. The strength of the correlations studied for which the cut-off values were as follows:

<0.2—no relationship between the studied variables;
0.2–0.4—weak dependence;
0.4–0.7—moderate dependence;
0.7–0.9—significant dependence;
0.9<—very strong dependence.

#### 2.3.3. Software

Statistical analyses were performed using Statistica 13.3.721.1 (TIBCO Software Inc., Palo Alto, CA, USA). Tables and graphs were prepared using Microsoft Office Excel 2007. Figures were prepared using Adobe Illustrator and VennPainter V.1.2.0 [31].

### 2.4. Ethics Statement

According to Polish law (the Experiments on Animals Act from 15 January 2015, Journal of Laws of the Republic of Poland from 2015, item. 266), this study did not require the Ethics Committee’s approval. The samples used in this study were originally from cattle infection diagnostic material collected by veterinarians treating these herds. Moreover, it did not cause the animals any pain, suffering, or distress greater than a needle-stick injury.

## 3. Results

### 3.1. Overall Results

A total of 4095 calves from 650 herds located in south-western Poland were examined. We found that 344/650 (52.92%) herds were seronegative, while in the remaining 306/650 (47.08%) herds, we found the presence of antibodies against at least one of the tested pathogens.

### 3.2. Seroprevalences

Antibodies against the following pathogens were detected in the examined herds: BoAHV1 in 140/650, BVDV in 208/650, BRSV in 222/650, BPIV3 in 223/650, and *M. bovis* in 204/650.

**Figure 2:**
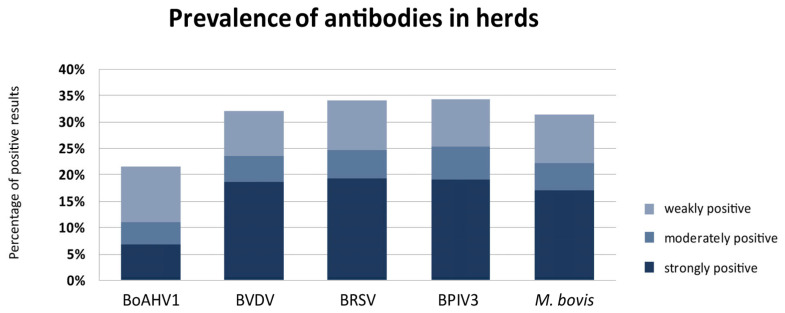
Overall seroprevalence for individual pathogens in the studied calf herds—divided by serum positivity rate.

The overall prevalence of antibodies was as follows: BoAHV1 21.54% (CI 95%: 18.55–24.86%), BVDV 32.0% (CI 95%: 28.53–35.68%), BRSV 34.15% (CI 95%: 30.61–37.88%), BPIV3 34.31% (CI 95%: 30.76–38.04%), and *M. bovis* 31.38% (CI 95%: 27.94–35.05%) (Figure 2).

Among the 306 positive results, the most frequently recorded sera had strongly positive SP: 18.62% (57/306) BVDV, 19.38% (59/306) BRSV, 19.08% (58/306) BPIV3, and 17.08% (52/306) *M. bovis*. In contrast, BoAHV1 sera had predominantly weakly positive SP 10.46% (32/306).

### 3.3. Configurations

The presence of antibodies against only one pathogen was recorded in 82 herds, representing 26.80% (82/306) of seropositive herds, and the most common were antibodies against BoAHV1 at 25.49% (78/306). Cooccurrence of sera against different pathogens was recorded in 224 herds, representing 73.20% (224/306).

The simultaneous presence of antibodies against two pathogens was recorded in 3.92% (12/306) of the herds, with the most frequently observed dual configuration being BRSV and PIV3, accounting for 2.94% (9/306).

A triple configuration was recorded in a total of 4.25% (13/306) of the herds, with the most common combination comprising antibodies against BRSV, BVDV, and BPIV3, accounting for 2.29% (7/306).

Quadruple configurations were observed in 46.73% (143/306) of the herds, with the most common being the concurrent presence of BVDV, BRSV, BPIV3, and *M. bovis*, which occurred in 46.08% (141/306) of the herds. This configuration was also the most frequently recorded result in the entire study.

A quintuple configuration was present in 18.30% (56/306) of seropositive herds, making it the third most frequently recorded result. All the obtained configurations are presented in Table 2.

**Figure 3:**
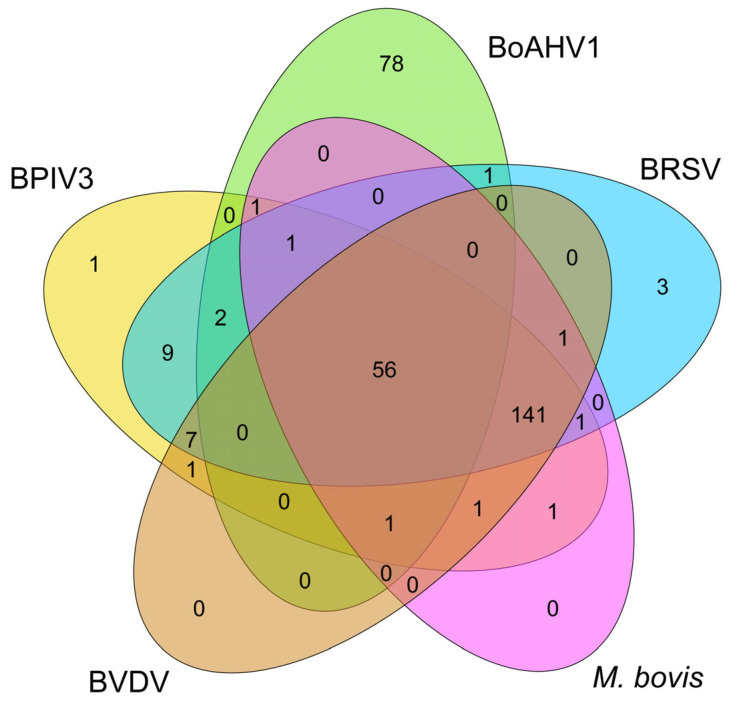
Venn diagram with all possible configurations of antibodies against BRSV, BPIV3, BoAHV1, BVDV, and *M. bovis*. The common parts indicate the types of configurations, while the numbers in the respective fields represent the frequency of each configuration.

Seventeen different configurations were recorded, although, with five factors examined, there were thirty-one possible configurations in total. All possible configurations and the frequencies of their occurrences in this study are presented in a Venn diagram (Figure 3).

### 3.4. Correlations

All correlations shown were positive. The strongest correlation was between BRSV positive sera and BPIV3 (r = 0.966) positive sera. The next-highest three correlation coefficients were for associations in which one of the studied traits was a positive serum against BVDV with *M. bovis* (r = 0.954), BPIV3 (r = 0.947), or BRSV (r = 0.933), respectively. No correlations were shown between the variables when one of the traits studied was the presence of antibodies against BoAHV1. Spearman’s rank correlation matrix for all the studied traits is presented in Table 3.

## 4. Discussion

### 4.1. Regional Conditions

To the authors’ knowledge, the last comprehensive survey in this region on the pathogens we studied in this age group of cattle was conducted more than a quarter of a century ago by Klimentowski et al. [22].

Since then, not only have there been technological developments but also significant economic and political changes like Poland’s accession to the European Union. These factors have facilitated the import of calves into Poland. There has also been a process of concentration of cattle breeding: the total number of farms in Poland has decreased but the density of cattle per pasture area has increased [32]. The introduction of calves into herds from other countries, their transport, and increased density may have contributed to a change in the seroprevalence structure of individual infectious agents responsible for BRD in calves [13].

Part of the European Union’s border is identical to much of the Polish border on the eastern side. However, the region we studied borders countries to the west and south that are also members of the European Union.

In the context of dairy cattle management, dairy herds are kept in two types of farms: free stalls, often with free access to paddocks, and tie stalls. Animals are usually kept on bedding of varying depths. Access to pasture is primarily found in tie–stall systems. Currently, the free-stall type prevails in the western part of Poland. In eastern Poland, tie-stall systems are more common, and many farms utilize access to pastures during the summer months [7,33].

### 4.2. Pathogen Reservoirs

The number of free-living European bison in Poland has grown considerably in recent years. As of the end of 2021, these herds collectively house 2223 bison, representing a unique situation on the European scale [34]. Klich et al. determined that in eastern Poland there is an increased risk of transmission of viral pathogens between European bison and breeding cattle due to the possibility of close contact between these animal species, mainly due to the sharing of pastures [35]. Moreover, the seroprevalence of pathogens responsible for BRD is higher among free-ranging European bison than those in isolated herds [36]. Other wild ruminants are probably not as important a reservoir as European bison; for example, Rola et al.’s studies have shown that it is highly unlikely that cervids in Poland could play any role in spreading BoAHV1 between cattle [37].

### 4.3. Seroprevalences

Seroprevalences for the individual pathogens that were the subject of this study vary between studies published from different centers around the world, and even between individual studies from a single country, depending on the year of the study, the region, the type of herd and group studied, and the study method used [10,11,23,38,39,40].

There are limited studies from Poland on the seroprevalence of the pathogens we present. Nevertheless, among these studies, BoAHV1 is the most extensively studied. A regional survey published by Klimentowski et al. reported a seroprevalence of 20.21% (113/559) among heifers and cows in south-western Poland [22]. This result is comparable to our finding of 21.54% (140/650) and that of Rypuła et al. in 2012, who reported a seroprevalence of 22.5% (172/763) among calves from the same age group and region as in our study [27]. Wernicki et al. examined calves of the same age but from eastern Poland and beef herds and determined the BoAHV1 seroprevalence to be 33.9% (39/115) [41]. Rola et al. noted a higher seroprevalence for this pathogen, reporting it as 49.8% (681/1366) among dairy cows from eastern Poland [42]. The most recent study, published in 2022, partially overlapping with our study region, reported an overall seroprevalence of 39.5% (117/296) [26].

The available data on BVDV seroprevalence among calves in Poland are more limited. Wernicki et al. conducted their study using the same test manufacturer as in our research, which allowed us to analyze their results based on the SP criteria we adopted. We evaluated their findings and determined that the seroprevalence among the calves in their study was 15.38% (12/78) [40]. In contrast, in a recent survey, Socha et al. recorded the highest seroprevalence for this pathogen, showing a rate of 50.3% (144/286) among calves [26]. Our study found herd-level seroprevalence of 32.0% (208/650). For comparison, earlier research by Klimentowski et al. in this region indicated an animal-level seroprevalence of 18.78% (105/559) [22].

The overall seroprevalence reported in two studies from 2013 and 2015 hovered around 60%. Studies from eastern Poland by Urban-Chmiel et al. revealed a BRSV seroprevalence of 60.0% (27/45) among calves, with a higher seroprevalence of 64.0% (32/50) determined for calves in the same region [28]. Similarly, Socha et al., who examined 324 dairy herds across Poland, reported an overall seroprevalence of 61.4% (1885/3070) among the animals studied. The seroprevalence in animals under two years of age was 54.9%, and in animals over two years of age, it was 66.1%, although the study did not provide the exact number of animals in these groups [43]. In comparison, Klimentowski’s 1995 study had shown a lower BRSV seroprevalence of 26.65% (149/559). In our study, the seroprevalence was 34.14% (222/650) [22].

Due to limited data availability, comparing data on BPIV3 seroprevalence among Polish cattle is challenging. The sole Polish study on BPIV3 seroprevalence, conducted by Klimentowski et al., indicated an overall seroprevalence of 49.19% (275/559) at the animal level [22]. Our study, covering the same region but conducted nearly thirty years later, found a herd-level seroprevalence of 34.31% (223/650).

Studies on *M. bovis* have shown seroprevalences among Polish calves of 47.8% (11/23) reported by Szacawa et al. and 64.3% (541/841) reported by Dudek et al., while in the region corresponding to our study, it was 60.3% (216/358) [23,44]. However, Bednarek et al. reported a seroprevalence of 89.4% (76/85) for this pathogen at the herd level [29]. These results differ significantly from our finding of 31.38% (204/650).

A recent study found that among the tested free-ranging European bison, seroprevalence was 66.7% (60/90) for BPIV3; 12.2% (11/90) for BRSV; 1% (3/308) and 6.99% (6/186) for BVDV; 0.6% (2/308) and 50.27% (90/179) for BoAHV1; and 3.33% (6/180) and 22.2% (6/27) for *M. bovis* [36,45,46,47]. The seroprevalence among bison is lower than that observed in farm cattle for BRSV, BVDV, and *M. bovis*. However, the seroprevalence for BPIV3 is higher in bison than the level found in calves in our study. Additionally, for BoAHV1, one study reported a higher seroprevalence in free-ranging bison than ours. In our opinion, this topic is intriguing and warrants further research.

### 4.4. Correlations and Coocurrences

The literature data from Poland on seroprevalence predominantly represent the presence of a single infectious agent causing BRD. Information on the simultaneous occurrence of antibodies to several pathogens and correlations between pathogens and all detected configurations of occurrences is less common, particularly in studies conducted in Poland. However, such data are available from other parts of the world. Comparing the results is challenging due to the different animal groups studied and the various pathogens examined [15].

**Figure 4:**
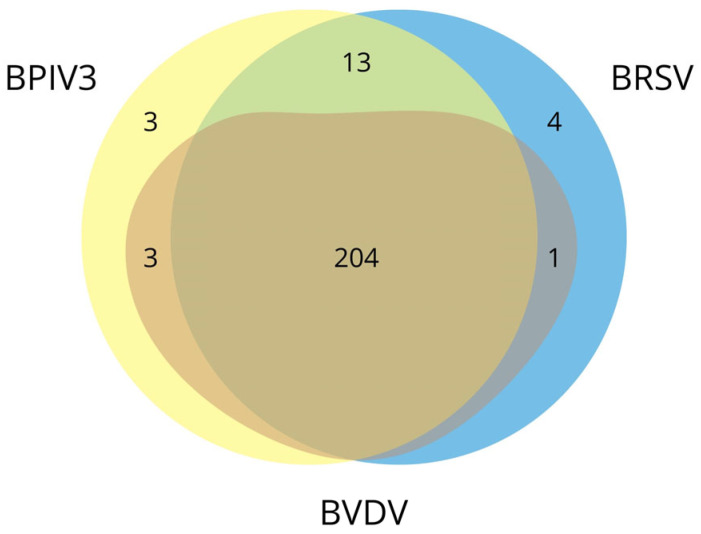
Euler diagram with the cooccurrences of BRSV, BPIV3, and BVDV recorded in this study, regardless of any additional antibodies present in these configurations. The common parts indicate the types of configurations, while the numbers in the respective fields represent the frequency of each configuration. BVDV did not occur independently.

Our study showed the strongest correlation for the pair BRSV and BPIV3 (r = 0.966). Chicoski et al. also identified correlations for the same pair among beef cattle but with a lower coefficient (ρ = 0.47) [48]. Nevertheless, it is noteworthy that this pair occurred alone in less than 2.94% (9/306) of positive results. BRSV, BPIV3, and BVDV configuration occurred in 2.29% (7/306). Despite the low percentage results, these configurations were the most frequently observed within their respective categories—those consisting of antibodies against two pathogens and those consisting of antibodies against three pathogens. BRSV, BPIV3, and BVDV configuration is the most common among cooccurrences; regardless of any additional antibodies present in these configurations, it was recorded in 66.67% of herds (204/306) (Figure 4).

It is worth noting that BVDV has the highest correlation with *M. bovis* (r = 0.954); however, presenting the data as sets revealed that it most frequently co-occurred with PIV3 and BRSV, with which it had lower correlations of (r = 0.947) and (r = 0.933), respectively (Figure 3). When antibodies *M. bovis* were additionally present, such a configuration (BVDV, BRSV, BPIV3, and *M. bovis*) was the most common positive result, recorded in 46% (141/306) of seropositive herds.

Similarly, in the study by Yoshitani et al., the BRSV and BPIV3 pair was noted in 3.0% (15/497) of results and was observed much more frequently when antibodies against other pathogens were present. The most common positive result was a combination of BoAHV1, BVDV, BPIV3, and BRSV, recorded in 44.1% (218/494) [15]. Similar results to those obtained by us were reported by Yousef et al. and Fulton et al., where the most common result was the joint occurrence of BRSV and BPIV3 antibodies: Yousef et al. reported 30.2% (139/460), while Fulton et al. found 35.6% (37/104). In their studies, this configuration extended with BVDV was the second most frequently recorded among results showing more than one type of antibody: Yousef et al. showed 11.30% (52/460), whereas Fulton et al. observed 18.3% (19/104) [49,50].

Hägglund et al. did not record the occurrence of the BRSV, BPIV3, and BVDV configuration as a standalone setup. However, these antibodies appeared together when those against BCoV were also present in 7.7% (1/13) of the first sample and 41.2% (7/17) of the second sample. Moreover, the BPIV3 and BRSV pair was observed in 13.3% (14/105) of cases in the first sample and 12.9% (13/101) cases in the second sample. [51]. Shirivani et al. presented the configurations of antibody cooccurrences among cattle in Iran for the viral agents that were included in our study [52]. Compared to our research, they did not note the simultaneous occurrence of antibodies to BRSV, PIV-3, and BVDV, even in configurations with other tested antibodies. In the referenced study, the most common configuration was BVDV with BoHAV1, which was recorded in 30.4% (174/573) [52]. Our study did not observe such a configuration as a single pair.

We found no statistically significant correlation between BVDV and BoAHV1 seropositivity, and this pair had the lowest correlation coefficient (r = 0.055). In contrast, a study conducted by Ghaemmaghami et al. among dairy herds in Iraq showed a statistically significant correlation between the presence of these antibodies in sera but it was nevertheless at a very low level (r = 0.192) [53]. For this association, Noaman et al. also found a statistically significant low correlation (r = 0.138) in their study of dairy cattle in Iran [54]. In our study, BoAHV1 was recorded with BVDV only when *M. bovis* was also present simultaneously, although the pair of BoAHV1 and *M. bovis* alone was not observed at all (Figure 3). In contrast to the mentioned studies, Chicoski et al. demonstrated a strong correlation for BoAHV1 with BPIV3 (ρ = 0.85). In our study, this association was not significantly correlated (r = 0.059) [48].

Socha et al.’s study showed that in sera collected from calves in Poland during 2014–2015, antibodies against BoAHV1 were present simultaneously with antibodies for BVDV in 30.3% (87/287) of cases, with BCoV in 33.5% (99/296), and antibodies against all three pathogens simultaneously in 25.1% (72/287); however, the study does not present the frequency of occurrence for configurations where antibodies against a single pathogen are detected. Additionally, BPIV3 was not included in the study [26]. Similar limitations are present in Gaetha’s study, which also did not present configurations of single occurrences and omitted BPIV3, making comparing our results more difficult. However, BoAHV1 and BRSV were the most frequently noted cooccurrences [55].

Considering how frequently configurations involving BoAHV1 occurred, we showed that antibodies to BoAHV1 were noted mainly as an independent result of 55.71% (78/140). Additionally, antibodies against BoAHV1 in our study mostly appeared weakly positive but high antibody titers against other tested pathogens were predominantly present in the tested sera. This may be due to the latency phenomenon and the BoAHV1 eradication program implemented among cattle in Poland [56,57]. More detailed research would be required on this issue. Similar results were reported in Gaeta et al.’s study, where antibody levels against BoAHV1 were recorded as low [55].

In contrast to our research, Yousef et al. showed that BoAHV1 was only detected in configurations where antibodies against multiple pathogens were present 100% (82/82) [49]. Similarly, Yoshitani et al. found that a significant majority of BoAHV1-positive sera were also positive for other pathogens 97.7% (347/355), while a single-factor configuration was recorded as positive in only 2.3% (8/355) [15]. In our study, the most frequently noted result was quadruple occurrences in 46.73% (143/306) of the herds. In Roshtkhari et al.’s study, triple occurrences were observed initially, followed by quadruple occurrences later; however, the exact combinations in which these occurred were not specified [58]. Gaeta et al. most frequently recorded double occurrences at 12.20% (15/123) while examining three types of pathogens [55]. Klimetowski et al.’s study from our region also indicated that the most frequently noted configurations were multiple pathogens, mainly double occurrences. However, this study did not provide detailed data on the number of occurrences for each specific configuration [22].

### 4.5. Limitations

In this study, the main limitation was that the test employed detected antibodies against only five known BRD-causing pathogens, whereas a wide range of infectious agents can cause BRD [8,9,10,11,12,13,14,15,16,17,18]. The calves examined in this study had previously shown signs of infection, indicating that they might have been exposed to pathogens not detected by the test. Subsequent molecular investigations conducted in the same region revealed the presence of other pathogens in symptomatic calves, such as BCoV, *P. multocida*, *M. haemolytica*, and *H. somni* [59].

It cannot be completely ruled out that some of the antibodies detected in this study were transmitted to calves with colostrums. Nevertheless, Fulton determined that the time for unvaccinated calves to become seronegative for the pathogens we tested ranged from 122.9 days (BoAHV1) to 192.2 days (BVDV) [24]. Similarly, the study by Tuncer et al. revealed the lowest seroprevalence for BPIV3 and BoAHV-1 in calves at six months of age, for BRSV at three months, and noted a decrease in BVDV seroprevalence in calves at four months. Additionally, the amount of antibodies can also depend on the individual characteristics of the organism in question. In the studies we conducted, serum samples from individual calves were pooled, which, among other things, aimed to reduce the impact of extreme results from individual calves on the overall test result for the herd. Therefore, the previous symptoms of BRD, the lack of specific prophylaxis in the form of vaccination, and the age of the calves studied (7–12 months) were expected to increase the chance that the antibodies detected in the study would be only those that the calves acquired during natural contact with the pathogen so that the results obtained would be most relevant to the age group examined.

Serological monitoring of the herd is a relatively inexpensive method. It allows for the assessment of what pathogens the animals in the studied herd have contacted in the past. Such knowledge makes it possible to plan anti-infective prophylaxis. Developing serological data for an entire region can help reduce the cost to farmers of having to order such tests in their herds and reduce animal stress by avoiding procedures that require taking samples.

## 5. Conclusions

The discrepancy in the data obtained worldwide regarding seroprevalence, correlation, and cooccurrence indicates the validity of conducting regional epidemiological studies. The statistical associations shown in this study between BRSV seropositivity and BPIV3, the frequent cooccurrence with antibodies to BVDV and *M. bovis*, and the high seroprevalence for these pathogens indicates the validity of carrying out preventive measures against these pathogens in south-western Poland.

## Figures and Tables

**Table 1:** Table for determination of serum positivity.

	0		+		++		+++		++++		+++++
BoAHV1	Val≤	30%	<Val≤	67%	<Val≤	104%	<Val≤	141%	<Val≤	178%	<Val
BVDV	Val≤	20%	<Val≤	40%	<Val≤	60%	<Val≤	80%	<Val≤	100%	<Val
BRSV	Val≤	23%	<Val≤	46%	<Val≤	68%	<Val≤	91%	<Val≤	114%	<Val
BPIV3	Val≤	26%	<Val≤	52%	<Val≤	78%	<Val≤	104%	<Val≤	130%	<Val
*M. bovis*	Val≤	29%	<Val≤	47%	<Val≤	64%	<Val≤	82%	<Val≤	100%	<Val

**Table 2:** Configurations of antibody cooccurrence in investigated cattle herds. The results are presented for the 306 herds where antibodies against at least one of the tested pathogens were found.

	Configuration		
No. of Pathogen-Specified Antibodies in Configuration	Pathogen’s Name	No. of Positive Results	% of All Positive Results
1	BPIV3	1	0.33%
	BRSV	3	0.98%
	BoAHV1	78	25.49%
2	BPIV3, *M. bovis*	1	0.33%
	BRSV, BPIV3	9	2.94%
	BVDV, BPIV3	1	0.33%
	BoAHV1, BRSV	1	0.33%
3	BRSV, BPIV3, *M. bovis*	1	0.33%
	BVDV, BPIV3, *M. bovis*	1	0.33%
	BVDV, BRSV, *M. bovis*	1	0.33%
	BVDV, BRSV, BPIV3,	7	2.29%
	BoAHV1, BPIV3, *M. bovis*	1	0.33%
	BoAHV1, BRSV, BPIV3,	2	0.65%
4	BVDV, BRSV, BPIV3, *M. bovis*	141	46.08%
	BoAHV1, BRSV, BPIV3, *M. bovis*	1	0.33%
	BoAHV1, BVDV, BPIV3, *M. bovis*	1	0.33%
5	BoAHV1, BVDV, BRSV, BPIV3, *M. bovis*	56	18.30%
At least one	-	306	100%

**Table 3:** Spearman’s rank correlation coefficient matrix between positive sera.

Correlations					
	BoAHV1	BVDV	BRSV	BPIV3	*M. bovis*
BoAHV1	1	0.055	0.0567	0.059	0.078
BVDV		1	0.933 *	0.947 *	0.954 *
BRSV			1	0.966 *	0.916 *
BPIV3				1	0.929 *
*M. bovis*					1

* Correlations statistically significant at *p* < 0.001.

## Data Availability

Data available on request from the corresponding author.
